# Heterologous Expression of Dehydration-Inducible *MfbHLH145* of *Myrothamnus flabellifoli* Enhanced Drought and Salt Tolerance in Arabidopsis

**DOI:** 10.3390/ijms23105546

**Published:** 2022-05-16

**Authors:** Zhuo Huang, Si-Han Jin, Li Yang, Li Song, Yuan-Hong Wang, Lin-Li Jian, Cai-Zhong Jiang

**Affiliations:** 1 College of Landscape Architecture, Sichuan Agricultural University, Wenjiang 611130, China; seraphimansuna@126.com (S.-H.J.); 2019310049@stu.sicau.edu.cn (L.Y.); imsongli@stu.sicau.edu.cn (L.S.); 201705768@stu.sicau.edu.cn (Y.-H.W.); jianlinli@stu.sicau.edu.cn (L.-L.J.); 2 Department of Plant Sciences, University of California Davis, Davis, CA 95616, USA; caizhong.jiang@usda.gov; 3 Crops Pathology and Genetics Research Unit, United States Department of Agriculture, Agricultural Research Service, Davis, CA 95616, USA

**Keywords:** *Myrothamnus flabellifolia*, resurrection plant, drought tolerance, Arabidopsis, basic helix–loop–helix (bHLH)

## Abstract

*Myrothamnus flabellifolia* is the only woody resurrection plant found in the world. It has a strong tolerance to drought and can survive long-term exposure to desiccated environments. However, few genes related to its drought tolerance have been functionally characterized and the molecular mechanisms underlying the stress tolerance of *M. flabellifolia* are largely unknown. In this study, we isolated a dehydration-inducible bHLH transcription factor gene *MfbHLH145* from *M. flabellifolia*. Heterologous expression of *MfbHLH145* enhanced the drought and salt tolerance of Arabidopsis. It can not only promote root system development under short-term stresses, but also improve growth performance under long-term treatments. Further investigation showed that *MfbHLH145* contributes to enhanced leaf water retention capacity through the promotion of stomatal closure, increased osmolyte accumulation, and decreased stress-induced oxidative damage through an increase in antioxidant enzyme activities. These results suggest that *MfbHLH145* may be involved in the positive regulation of stress responses in *M. flabellifolia*. This study provides insight into the molecular mechanism underlying the survival of *M. flabellifolia* in extreme dehydration conditions.

## 1. Introduction

The whole life of plants is threatened by a range of abiotic stresses, which can lead to irreversible damage. To survive the changing environments, plants have evolved sophisticated mechanisms to regulate their responses to stresses, which are controlled by complex regulating networks involving a wide range of genes [1]. Among these, transcription factors (TFs) play pivotal roles in strengthening the plant’s resistance to adverse conditions [2]. 

The basic helix–loop–helix (bHLH) superfamily is a large group of transcription factors defined by the bHLH signature domain. The basic region in a bHLH protein is usually composed of approximately 15 basic amino acid residues at the N-terminus and mainly functions in protein–DNA interaction, whereas the HLH region is composed of 50–60 amino acids and bears two amphipathic α-helices linked to each other by a loop motif (with variable amino acids) and is involved in the formation of protein complexes, such as homodimers or heterodimers, through protein–protein interaction. These domains are able to bind E- boxes (CANNTG) [3,4,5,6].

The bHLH family has been widely studied in eukaryotic lineages [7]. For example, there are 225 members of the bHLH family proteins in Arabidopsis, 211 members in rice, and 308 members in maize [8]. Previous functional characterizations have indicated that bHLH TFs play critical roles in regulation of various physiological processes, such as stomata development [7], flowering regulation [9], trichome or root hair development [10,11], chloroplast development [12], nodule vascular patterning [13], and photo-induced signaling transduction [14]. Furthermore, recent research has indicated that bHLH transcription factors are also involved in the regulation of adverse stress responses. Several Arabidopsis bHLH genes, such as *AtbHLH38*, *AtbHLH39*, *FER-LIKE IRON DEFICIENCY-INDUCED TRANSCRIPTION FACTOR* (*FIT*), *AtbHLH100*, and *AtbHLH101*, exhibit induced expression by which to mediate iron acquisition and regulate plant detoxicity to heavy metal stress [15,16,17]. *AtbHLH122* is important for drought and osmotic stress resistance in Arabidopsis [18]. In response to drought stress, *GhbHLH1* is suggested to function in the ABA signaling pathway [19]. The *Populus euphratica* gene *PebHLH35* confers drought tolerance by controlling stomatal aperture and closure in leaf [20,21]. Overexpression of *VvbHLH1* of *Vitis viniferain* in Arabidopsis enhanced tolerance to salt and drought [22]. *OsbHLH148* of rice is involved in drought tolerance [23], and wheat *TabHLH1* is crucial in mediating osmotic stress tolerance through large modulation of the ABA-associated pathway [24]. Therefore, the bHLH TF family could be considered as a reservoir of genes essential for abiotic stress tolerance.

*Myrothamnus flabellifolia* is a woody homoiochlorophyllous resurrection plant distributed in the mountainous regions of central and southern Africa [25,26]. The molecular mechanisms underlying the tolerance of *M. flabellifolia* to extreme drought conditions and its ability to rapidly rehydrate are still largely unknown. Ma et al. (2015) performed transcriptome analysis of *M. flabellifolia* during dehydration and found that many TFs (295) were responsive to dehydration [27]. The MYB (MYB proto-oncogene), WRKY, and bHLH families were among the largest groups during both dehydration and rehydration, in which at least eight unigenes encoding putative bHLH TF were upregulated at early stage of dehydration. In this study, one of the unigenes, *comp43792_c0_seq1* was cloned and characterized. To further investigate its functions in stress responses, it was overexpressed in model plant Arabidopsis. The enhanced drought and salt tolerances in transgenic plants were found and their potential roles involved in stress response regulation were further characterized and discussed.

## 2. Results and Discussions

### 2.1. Isolation and Sequence Analysis of MfbHLH145

**Figure 1:**
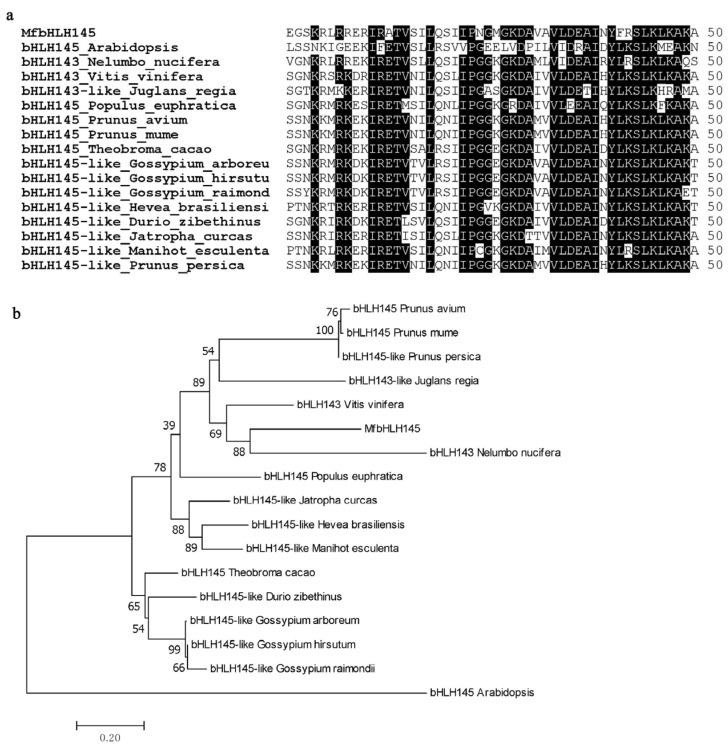
Comparison of MfbHLH145 and its homologous sequences. (**a**) Comparison of HLH domain; (**b**) phylogenetic relationship between MfbHLH145 and bHLH proteins from other plant species. The species and accession numbers for the sequences used are as follows: *Arabidopsis* (AT5G50010.1), *Nelumbo nucifera* (XP_010272947.2), *Vitis vinifera* (XP_010664370.1), *Juglans regia* (XP_018833850.1), *Jatropha curcas* (XP_012067965.1), *Populus euphratica* (XP_011012705.1), *Prunus avium* (XP_021808181.1), *Prunus mume* (XP_016647743.1), *Prunus persica* (XP_007221781.1), *Theobroma cacao* (XP_007018176.2), *Gossypium arboreum* (XP_017606889.1), *Gossypium hirsutum* (XP_016748867.2), *Gossypium raimondii* (XP_012445605.1), *Hevea brasiliensis* (XP_021684509.1), *Manihot esculenta* (XP_043810156.1), *Durio zibethinus* (XP_022773104.1). Phylogenetic reconstruction was performed using the neighbor-joining approach, and the bootstrap test was replicated 1000 times.

The obtained cDNA of unigene *comp43792_c0_seq1* gene is 1020 bp in length (Figure 1a), which contains a complete open reading frame (ORF) encoding 339 amino acids, with predicted molecular mass of 37.42 kD and pI (isoelectric point) of 4.83 (Figure S1). We performed a blastp search against Araport11 whole genome protein sequences of Arabidopsis (www.arabidopsis.org, (accessed on 5 March 2022)) and found that it showed the highest homology to bHLH145 of Arabidopsis (AT5G50010.1). Thus, we designated it as *MfbHLH145*. Multiple alignment of amino acid sequences with several most homologous sequences (obtained by blastp against NCBI nr database) and subsequent phylogenetic analysis indicated that they all contain a highly conserved HLH domain (Figure 1a, Figure S1) and share high similarity to the bHLH family members derived from various plant species, such as *Nelumbo nucifera*, *Vitis viniferap*, *Populus euphratica*, *Jatropha curcas*, *Corchorus capsularis*, etc. (Figure 1b). These results indicate that the *MfbHLH145* belongs to bHLH family.

### 2.2. MfbHLH145 Is Localized in the Nucleus

**Figure 2:**
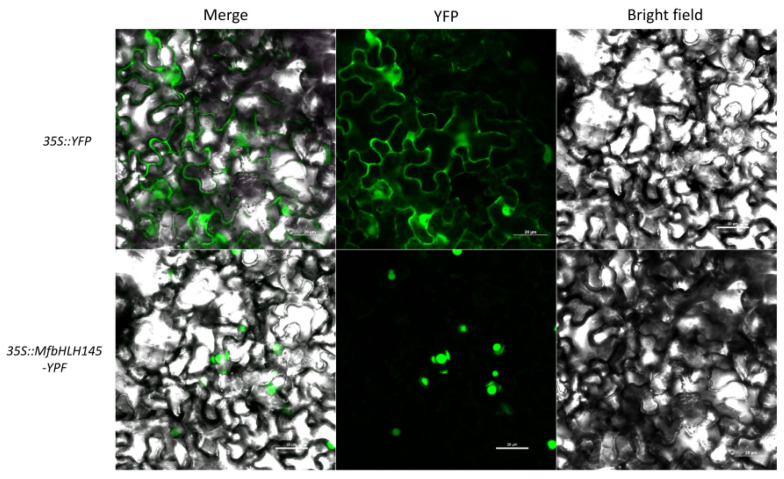
Subcellular localization of MfbHLH145. Upper: Fluorescence detection of tobacco epidermis transformed with 35S:YFP; Lower: Fluorescence detection of MfbHLH145-YFP fusion protein in tobacco leaf epidermal cells. Scale bar indicates 20 μm.

A nuclear localization signal site (PSKKRKLSL) was found at position 241 of the deduced protein of MfbHLH145 (Figure S1), and no nuclear export signal was found by using LocNES [28]. We then investigated the subcellular localization of MfbHLH145 based on observation of the YFP signal derived from MfbHLH145-YFP fusion in the tobacco epidermal cells. Fluorescence from 35S-YFP was detected in the cytoplasm and nucleus, whereas fluorescence from the 35S-MfbHLH145-YFP fusion was detected only in the nucleus (Figure 2), which was consistent with in silico prediction and indicated that MfbHLH145 may function as a TF.

### 2.3. Overexpression of MfbHLH145 in Arabidopsis Enhanced Tolerance to Drought and Salt

**Figure 3:**
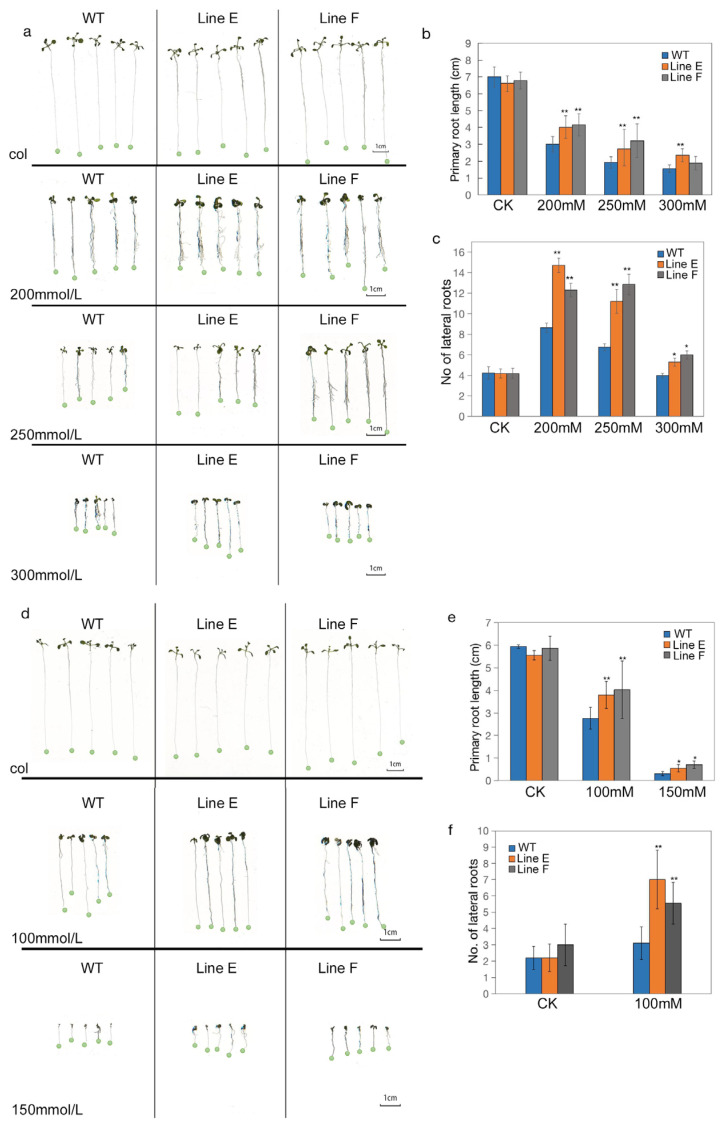
Measurement of tolerance to drought and salt stresses at seedling stage. (**a**) The performance of WT and transgenic Arabidopsis seedlings under artificially simulated drought stress. Seeds were germinated on 1/2 MS culture medium and then were transferred to 1/2 MS culture medium containing mannitol (0 mM, 200 mM and 250 mM, 300 mM) for 7 days. (**b**,**c**) Comparison of the length of primary roots and number of lateral roots among transgenic lines and WT under drought treatment. (**d**) The performance of WT and transgenic Arabidopsis seedlings under salt stress. Seeds were germinated on 1/2 MS medium and then were transferred to 1/2 MS medium containing NaCl (0 mM, 100 mM and 150 mM) for 7 days. (**e**,**f**) Comparison of the primary root length and number of the lateral roots of transgenic lines and WT under salt stress. Mean values and standard errors (bar) are shown from three independent experiments. The asterisk represents significant difference (**, *p* < 0.01). col in (**a**,**d**), and CK in (**b**–**f**) indicate growing conditions without stress treatment (control).

Although some bHLHs have confirmed involvement in regulation of biotic stress responses, the functions of bHLHs are largely unknown, and the roles of bHLH145s are unclear. As MfbHLH145 is inducible by dehydration, whether it participates in stress response regulation is an interesting question. We generated transgenic lines of Arabidopsis overexpressing *MfbHLH145*. To investigate the effect of drought stress on seedling growth, seeds of the two transgenic lines, line E and line F, and wild type (WT) were sowed on MS medium without (control) or with different concentrations of mannitol (200 mM, 250 mM, and 300 mM). WT and transgenic lines showed similar growth on control medium. Under the mannitol treatments, the primary root growth of WT and transgenic lines were significantly inhibited. However, line E and line F exhibited significantly longer primary roots than those of WT (Figure 3a,b). The numbers of lateral roots of WT and transgenic seedlings were increased under 200 mM and 250 mM mannitol treatments. However, both transgenic lines exhibited significantly more lateral roots than that of WT (Figure 3a,c). Under treatment of 300 mM mannitol, the lateral root numbers of WT and transgenic lines E and F were significantly less than those of 200 mM and 250 mM mannitol treatments. However, they both had more lateral roots than that of WT. These results indicate that MfbHLH145 could promote development of a root system under drought. In maize, overexpression of a bHLH gene *ZmPTF1* could increase root length and number of lateral roots under normal and stressful conditions [29]. However, in our study, lines overexpressing MfbHLH145 showed similar root growth with WT under normal condition. This result indicates that MfbHLH145 indirectly promoted root growth under drought stress.

We further investigated seedling growth performance under salt stress. 100 mM NaCl significantly inhibited primary root growth. Whereas line E and line F showed longer primary root length than that of WT (Figure 3d,e). On the other hand, they also exhibited higher a number of lateral roots than those of WT under 100 mM NaCl, as well as those of all three lines grown on control medium (Figure 3b,f). Under 150 mM NaCl, root growth of all three lines was severely inhibited. Line E and line F still showed longer roots than WT (Figure 3e).

**Figure 4:**
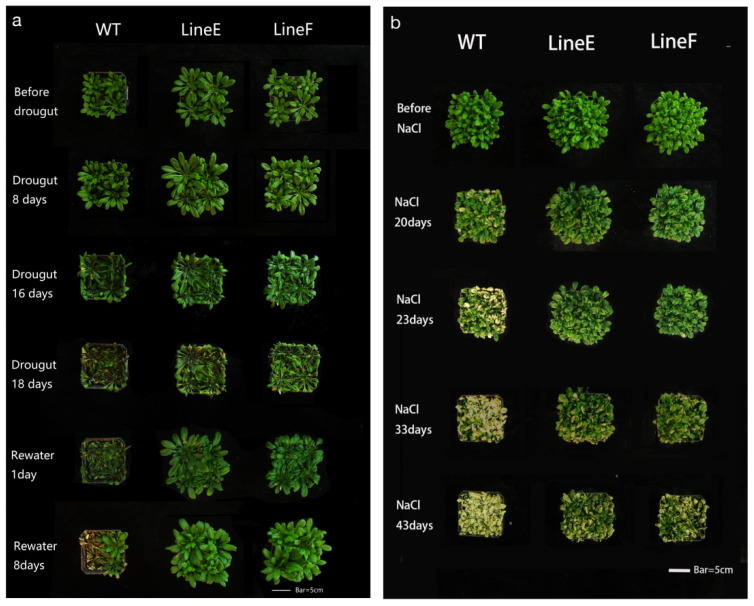
The performance of adult WT and transgenic plants under natural drought (**a**) and salt treatments (**b**). WT, wild type. The pictures show the same pots photographed at different time points. All experiments were repeated three times. Scale bar represented 5 cm.

To further evaluate growth performance of adult plants under both types of stresses, four-week-old plants were treated by natural drought stress and salt treatment. As shown in Figure 4a, the dehydration symptoms could be observed on leaves after withholding water for 8 days. After 16 days, significantly negative effects of drought on plant growth were found. All the plants were significantly wilted. WT plants were more severely wilted than line E and line F. After 18 days, the degree of leaf wilting of both WT and transgenic lines continued to increase. Leaves of most of the WT plants were bleached, whereas major part of leaves of transgenic lines stayed green. After rewatering, line E and line F recovered more quickly than the WT plants did. Eight days after rewatering, about 50% of WT plants died, whereas both transgenic lines returned to normal growth level.

Similar results were found in salt treatment. Four-week-old plants in each pot were watered with 600 mL 300 mM NaCl solution. No significant difference was found between WT and transgenic lines before treatment. After 20 days of treatment, some leaves of WT and lines E and F were withered. After 23 days, significant leaf bleaching and wilting were found in WT, and chlorisis was present in the line E and line F. After 33 days, almost all WT leaves were bleached and wilted. Although all leaves of the transgenic lines were wilted, only a few bleach symptoms was found. After 43 days, all WT plants were dead, whereas green leaves could be clearly found in transgenic lines (Figure 4b). All these results confirm that overexpression of *MfbHLH145* can enhance tolerance to both drought and salt stresses in Arabidopsis, suggesting that MfbHLH145 may function positively in regulation of stress response in *M. flabellifoli*. bHLH145 belongs to subfamily 13 of bHLH family in Arabisopsis [5], on which less attention has been paid. Rice bHLH142 plays a crucial role in pollen development [30], bHLH144 has been reported to be involved in activation of Wx to regulate grain quality in rice [31]. The functions of bHLH143 and bHLHL145 were previously unknown. Thus, MfbHLH145 is the first member of subfamily 13, which participates in response to drought and salt stresses.

### 2.4. MfbHLH145 Enhanced Leaf Water Retention Capacity under Drought and Salt Stresses

**Figure 5:**
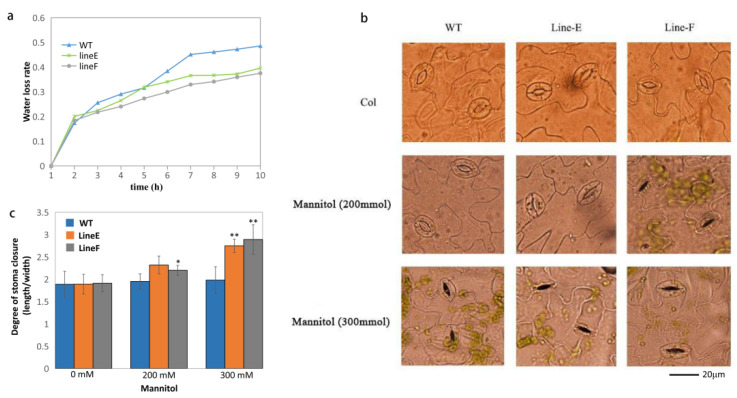
Evaluation of water retention ability. (**a**) Dynamic water loss rates of detached leaves. About 0.5 g leaves from plants in similar growth state were cut off and used for measurement of water loss rates. The experiments were performed with three replicates. (**b**,**c**) Stomatal closure in response to artificially simulated drought conditions (200 mM and 300 mM mannitol). Mean values and standard errors (bar) are shown from more than 100 stomata. The asterisk represents significant difference (*, *p* < 0.05; **, *p* < 0.01). Col, control.

In response to drought or osmotic stress, plants are able to control their water content and reduce water loss [32]. To further investigate the function of MfbHLH145 in responding to abiotic stress, we compared the water retention capacity between the WT and *MfbHLH145* overexpression lines. Water loss rate is dependable for assessing plant water status under drought stress [33]. The measurement of water loss from detached leaves of WT and transgenic plants Line E and Line F showed that the plants overexpressing *MfbHLH145* lost water more slowly than the WT after dehydration for 3 h (Figure 5a). After 7 h, the water loss rate of the WT plants was approximately 49%, in comparison, the water loss rates were ~40% and ~38% in Line E and Line F, respectively. 

According to a previous study, water loss mainly depends on stomatal regulation [34]. We then compared stomatal apertures under simulated drought treatments with different concentrations of mannitol (0 mM, 200 mM, and 300 mM). No significant difference of stomatal aperture was found under stress-untreated conditions between the WT and transgenic lines. By contrast, after mannitol treatments, especially with 300 mM concentration, transgenic lines showed a significantly higher degree of stomatal closure than that of WT, which was not obviously altered (Figure 5b).

Calculation of the Stomatal Aperture Index (SAI) showed that under 300 mM mannitol, the SAIs of Line E and Line F were about 2.7 and 2.9, respectively, significantly higher than that of WT (~2.0) (Figure 5c). These results indicate that transgenic lines closed stomata more quickly and tightly under drought stress, which explain, at least partly, the lower water loss rate in *MfbHLH145* overexpression lines. Ectopic expression of apple *MdbHLH130* in tobacco showed significantly lower water loss rate and stomatal aperture ratio (width/length), resulting in improved tolerance to water deficit stress [35]. This suggests that *bHLH145* and *bHLH130* may share a similar function in responding to drought stress.

### 2.5. MfbHLH145 Increased Osmolytes Accumulation under Drought and Salt Stresses

**Figure 6:**
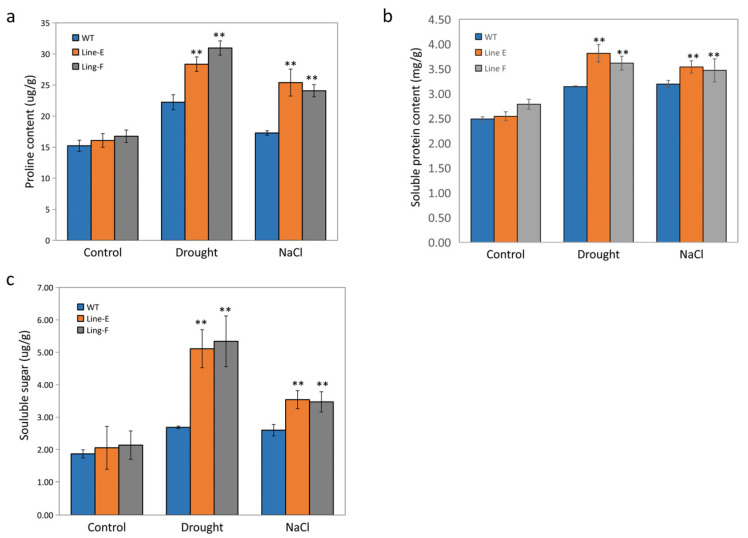
Measurement of content of free proline (**a**), soluble protein (**b**) and soluble sugar (**c**) in WT and transgenic lines under drought and salt treatments. Mean values and standard errors (bar) are shown from three independent experiments. The asterisk represents significant difference (**, *p* < 0.01). For each experiment, about 0.5 g leaves from each line were sampled from more than five individual plants with similar growth.

Osmotic stress is the earliest challenge of plants under drought conditions [36]. Proline is an important osmolyte in plants and is considered one of the compatible osmolytes in combating/ameliorating the detrimental effects of drought stress in many plants [37]. We measured free proline content in the WT and transgenic plants under normal and stress conditions. Under normal conditions, the WT and both transgenic lines showed no difference in proline content. However, line E and line F accumulated significantly more free proline under both the drought and salt stress conditions (Figure 6a). We also measured two other major osmolytes: soluble proteins (SP) and soluble sugar (SS) [38]. Similar to the results of the proline content, transgenic lines also showed significantly higher SP and SS accumulations under stress conditions comparing to WT (Figure 6b,c). These results indicate that overexpression of *MfbHLH145* increased accumulation of osmotic substances to adjust responses to osmotic stresses caused by drought and salt treatments. Several bHLHs were reported to play roles in osmotic stress tolerance. bHLHL92 is NaCl-induced and confers tolerance to salt and osmotic stress which is partially dependent on ABA and SALT OVERLY SENSITIVE 2 (SOS2) [2]. Overexpression of *AtbHLH122* confers salt tolerance, osmotic-regulating capacity, and proline concentration [18]. The increased osmotic regulatory ability of transgenic Arabidopsis could be also obtained by overexpression of *MfbHLH38*, a *Myrothamnus flabellifolia* bHLH transcription factor [39]. Thus, enhancement of osmotic-regulating capacity is a general function for bHLHs responsive to abiotic stress. How MfbLHL145 regulates the accumulation of osmotic substances in responding to drought and salt stresses deserves further study.

### 2.6. MfbHLH145 Decreased Stress-Induced Oxidative Damage through Increasing Antioxidant Enzyme Activities

**Figure 7:**
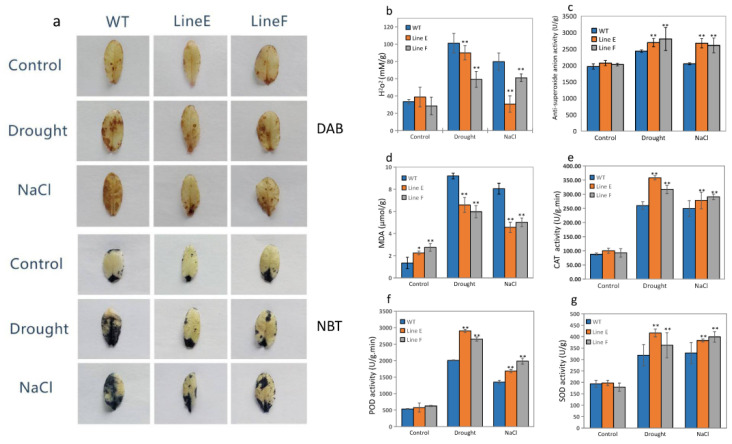
Evaluation of ROS-induced damage and ROS-scavenging ability. (**a**) DAB staining and NBT staining of leaves of WT and transgenic lines under normal and stress conditions. (**b**–**d**) showed H_2_O_2_ content, anti-superoxide anion activity, and MDA content, respectively; (**e**–**g**) indicated measurement of the activities of antioxidant enzymes. All treatments and measurements were performed three times. Mean values and standard errors (bar) are shown from three independent experiments. The asterisk represents significant difference (*, *p* < 0.05; **, *p* < 0.01). DAB and NBT indicate leaves stained by 3,3′-diaminobenzidine and nitroblue tetazolium, respectively.

Plants are usually injured from ROS-associated damage caused by environment stress [40,41]. We then investigated ROS levels in WT and transgenic lines under drought and salt stresses by evaluating accumulation of two major ROS species, H_2_O_2_ and superoxides. By using 3,3′-diaminobenzidine (DAB) and nitroblue tetazolium (NBT) staining, as shown in Figure 7a, we found that WT and *MfbHLH145* overexpression lines showed similar and low levels of H_2_O_2_ and superoxides before treatment. However, after exposure to drought and salt treatments, more H_2_O_2_ and superoxide accumulations were detected in WT compared to the two transgenic lines. These results are consistent with measurement of H_2_O_2_ content and anti-superoxide anion activity in leaves under drought and salt treatments (Figure 7b,c).

The Malondialdehyde (MDA) content is a reflection of lipid peroxidation and is usually used to measure stress-induced damage [38]. Under normal conditions, WT and transgenic lines E and F showed low levels of MDA content, although MDA levels in two transgenic lines were slightly higher than that of WT. After treated by drought and salt stresses, MDA levels in WT and transgenic lines remarkably increased. However, both line E and line F exhibited significantly lower levels of MDA compared with those of WT (Figure 7d). These results indicate that overexpression of *MfbHLH145* protected plants from stress-induced oxidative damage.

To further uncover potential mechanisms underlying decreasing oxidative damage in *MfbHLH145* overexpression lines, we measured the activities of antioxidant enzymes, i.e., catalase (CAT), peroxidase (POD) and superoxide dismutase (SOD). Our results indicate that under normal conditions, activities of three enzymes were similar in WT and transgenic lines. After exposure to drought and salt stresses, dramatically increased activities of CAT, POD, and SOD were found in WT, line E and line F. However, those of transgenic lines were significantly higher than those of WT (Figure 7e,f). These results show that overexpression of *MfbHLH145* increased ROS-scavenging ability and resulted in decreased stress-induced oxidative damage under drought and salt stresses.

## 3. Conclusions

In this study, we isolated and characterized a dehydration-inducible bHLH transcription factor gene *MfbHLH145* from *M. flabellifolia*, the only woody resurrection plant in the world. Heterologous expression of *MfbHLH145* in Arabidopsis enhanced tolerance to drought and salt. Our results show that *MfbHLH145* can not only promote root system development under short-term stresses, but also improve growth performance under long-term treatments. *MfbHLH145* contributes to enhanced leaf water retention capacity through promotion of stomatal closure, increased osmolytes accumulation, and decreased stress-induced oxidative damage via increased antioxidant enzyme activities. All these data suggest that *MfbHLH145* may be involved in positive regulation of abiotic stress responses in *M. flabellifolia*. No study has reported involvement of bHLH145 in responding to drought and salt stress previously. Therefore, bHLH145 has potential application value in the genetic improvement of plant stress tolerance. Further work on how it works at a molecular level is necessary for better understanding of the molecular mechanism underlying the survival of *M. flabellifolia* from extremely dehydration conditions.

## 4. Materials and Methods

### 4.1. Plant Materials and Growth Conditions

The Arabidopsis ecotype Columbia (Col) is conserved by our lab. The seeds were surface-sterilized with 50% bleach for 5 min, and washed with sterilized water three times. The sterilized seeds were sowed on one-half-strength Murashige and Skoog (MS) medium (supplemented with 3% sucrose and 0.8% agar and adjusted pH to 5.8) plates. To break dormancy, seeds were incubated at 4 °C for two days in the dark before germination and then placed in a plant growth incubator (22 °C, 16 h light/8 h dark cycle). After one-week growth in the incubator, seedlings were transferred into soil in a growth chamber at 22 °C under 16 h light/8 h dark cycle.

### 4.2. Gene Cloning and Sequence Analysis 

Total RNA was extracted from leaves of *M. flabellifolia* using Plant Total RNA Isolation kit (TIANGEN Co., Beijing, China) and cDNA synthesis was performed by Reverse Transcriptase M-MLV (RNase H-) (TaKaRa Bio, Dalian, China). The coding sequence of *MfbHLH145* gene was amplified by PCR with gene-specific primers containing NcoI and SpeI (forward: 5′-CATGCCATGGGAAAGGACTGTGGATCC-3′, and reverse: 5′-GCTAGTGAGAGAATCAAGTCCTAAAGCTTTG-3′) and then cloned into pEasy-T1 Simple vector (TransGen Biotech, Beijing, China). The constructs were transformed into the *E. coli* strain DH5α, and three positive clones were randomly selected and sequenced by commercial company (TsingKe, Beijing, China).

The SMART (http://smart.embl-heidelberg.de/ (accessed on 5 March 2022)) was used to predict the domain of gene structure. The BLAST program was used to search NCBI nr database for homologues in other plant species and the putative functional domains in the deduced amino acid sequence [42]. The multiple sequence alignment and phylogenetic analyses were performed using MEGA 7 [43].

### 4.3. Subcellular Localization of MfbHLH145 

By using the primers FP (ACCAGTCTCTCTCTCAAGCTTATGGGAAAGGA-CTGTGGATCC), and RP (GCTCACCATACTAGTGGATCCGAGAGAATCAAGTCCTA-AAGCTTTG), the cDNA fragment containing the *MfbHLH145* coding region without stop codon was cloned and inserted downstream of the Cauliflower Mosaic virus (CaMV) 35S promoter and in frame with the 5′ terminus of the YFP gene in the PHB vector. 

The plasmid construct 2*35S:MfbHLH145:YFP was transformed into *Agrobacterium tumefaciens* strain GV3101 through freeze-thaw method and the Agrobacterium-mediate transformation into *Nicotiana benthamiana* was conducted by impregnation method. To examine the subcellular localization of MfbHLH145, the 2*35S:MfbHLH145:YFP fusion protein was observed using a confocal laser scanning microscope (LSM510 META, Zeiss, Jena, Germany). 

### 4.4. Generation of Transgenic Plants 

The amplified cDNA fragment of coding region was digested by NcoI and SpeI and ligated onto pGSA1403 driven by the CaMV 35S promoter. The recombinant vector 35S:MfbHLH145:pGSA1403 was transformed into *A. tumefaciens* strain LBA4404 and then used to generate transgenic Arabidopsis plants with the floral dip method [44]. In order to screen positive transgenic lines, approximately 100 seeds were placed on 1/2 MS medium containing 3% sucrose and 0.8% agar and 100 μg/mL Kanamycin. The seeds were treated under 4 °C for two days in the dark and transferred into an incubator (22 °C, 16 h light/8 h dark cycle). Dark green plants were transferred into soil after a week to harvest seeds. The positive transgenic plants were identified by PCR, and two homozygous T_3_ lines, ling E and line F, were randomly selected for further investigation.

### 4.5. Drought and Salt Treatment

For seedling drought and salt treatments, sterilized seeds of wild type (WT) and T_3_ transgenic lines E and F were sown on square plates containing solid 1/2 MS medium (supplemented with 3% sucrose and 0.8% agar and adjusted pH to 5.8) with different concentrations of mannitol or MS medium with different concentrations of NaCl, respectively. After two-days incubation at 4 °C in dark, the seeds were vertically grown in an incubator (22 °C, 16 h light/8 h dark cycle), and the root length and the number of lateral roots were recorded after two weeks. For each sample, 15 seedlings in every petri dish was measured and each experiment was performed in three replicates.

To evaluate tolerance of adult plants to drought and salt stresses, seeds of WT and T_3_ transgenic line E and line F were germinated on 1/2 MS medium in normal condition, and then transferred to 10cM pot filled with peat soil and grown under normal condition for four weeks. The natural drought treatment was performed. The four-week-old plants of WT, line E and line F were firstly well watered and then the water was withheld. To perform salt treatment for the adult plants, 300 mM NaCl solution was applied in the tray of cultivation pots for irrigation, and to keep the soil moist during the processing. All experiments were repeated three times. The morphological changes of the plants were constantly observed and photographed.

### 4.6. Assays of Water Loss and Stomatal Aperture Closure

To determine the water loss rate, leaves from five-week-old WT and transgenic plants (about 0.5 g leaves from plants in similar growth state) were cut off and weighed immediately on a piece of weighting paper, then dried on 3 mm filter paper at room temperature (40% relative humidity) and weighed at designated time points. The percentage loss of fresh weight was then calculated. The experiments were performed with three replicates. 

For stomata aperture measurements, WT and transgenic plants were grown on soil under normal conditions for four weeks. Rosette leaves were detached and floated for 2 h in MES-KCl solution containing 50 mM KCl and 10 mM MES (pH 6.15), and a cool white fluorescent light was employed to induce maximum stomata opening. For measurements under simulated drought stress, leaves from WT and transgenic plants were floated in MES-KCl solution with additional 300 mM mannitol. Stomatal aperture was scored as width/length pore ratio of at least 100 stomata (*n* = 100).

### 4.7. Measurement of Biochemistry Parameters Related to Stress Responses

Plants were grown under normal conditions for four weeks. The samples without treatment were used as control. Then, the plants were watered with 300 mM NaCl solution for 2 days or exposed to drought by withholding water for 14 days. For the proline and soluble protein content measurements, about 0.5 g of leaves from each line were sampled from more than five individual plants with similar growth. The extraction and measurement were conducted according to previous report [34]. The soluble sugar was measured using Plant Soluble Saccharide Assay Kit (Nanjing Jiancheng Bioengineering Institute, Nanjing, China). 

Accumulation of malondialdehyde (MDA) and activities of three major ROS-scavenging enzymes, superoxide dismutase (SOD), catalase (CAT), and peroxidase (POD) were measured according to methods used in previous work [38]. The content of H_2_O_2_ was measured using Hydrogen Peroxide Assay Kit (Nanjing Jiancheng Bioengineering Institute, Nanjing, China), and Anti-superoxide Anion Activity was measured using Anti-superoxide Anion Activity Assay Kit (Nanjing Jiancheng Bioengineering Institute, Nanjing, China). All experiments were repeated three times.

## Data Availability

Not applicable.

## Supplementary Materials

The following supporting information can be downloaded at: https://www.mdpi.com/article/10.3390/ijms23105546/s1.
