# Supplementary material for: Heterologous Expression of Dehydration-Inducible MfbHLH145 of Myrothamnus flabellifoli Enhanced Drought and Salt Tolerance in Arabidopsis
Source: Int J Mol Sci. 2022 May 16;23(10):5546. doi: 10.3390/ijms23105546 (PMC9146472; doi:10.3390/ijms23105546)
Supplement: Supplementary file 1 [file ijms-23-05546-s001.zip › ijms-1691014-supplementary.pdf]

```
                10        20        30        40        50        60        70
1        ATGGGAAAGGACTGTGGATCCTGGTTTCCCCAGCGGCATTATTGTGAGCAATCACCCGATCTGGATAGGTTGTGT
1         M  G  K  D  C  G  S  W  F  P  Q  R  H  Y  C  E  Q  S  P  D  L  D  R  L  C

                85        95       105       115       125       135       145
76       CCTCCCCTTCATTTGGGACAGCAAAATATGATCCCATCCTACATGAACCCTTACTCTAATTCGGTTTCTACATAT
26        P  P  L  H  L  G  Q  Q  N  M  I  P  S  Y  M  N  P  Y  S  N  S  V  S  T  Y

               160       170       180       190       200       210       220
151      GGGATTTTGCCAATGTTACAATCTCCTGAGTCTCTCCATTTGACGGCAAACCAAGCGGATGAACCTCATGGATGG
51        G  I  L  P  M  L  Q  S  P  E  S  L  H  L  T  A  N  Q  A  D  E  P  H  G  W

               235       245       255       265       275       285       295
226      TTTTATTGTTTGCCCCATCATCGCCAGGCTATTTCCCCTGGATCGAAGTCAGTTATCAAAGATGAAGTTCCTTCT
76        F  Y  C  L  P  H  H  R  Q  A  I  S  P  G  S  K  S  V  I  K  D  E  V  P  S

               310       320       330       340       350       360       370
301      AGCCTTTATAAAGCAGATACAGATCAGAGGAGGTTCTTAGTTTTTGATCAATCTGGCGATCAAACAACCTTCATG
101       S  L  Y  K  A  D  T  D  Q  R  R  F  L  V  F  D  Q  S  G  D  Q  T  T  F  M

               385       395       405       415       425       435       445
376      TTCAGTCCAGCTATTAGGACTTCCATGGAGGGCCTGAGTTCTTTTGGTCCAAGTCCTTATAATGGTCATAACTTT
126       F  S  P  A  I  R  T  S  M  E  G  L  S  S  F  G  P  S  P  Y  N  G  H  N  F

               460       470       480       490       500       510       520
451      AGTGGGGAAAGGCCAGGTACTGAAAGAGACTCGCTTCACAATGCTGGTCCTATTCTAACAGATGGAAACGAAATT
151       S  G  E  R  P  G  T  E  R  D  S  L  H  N  A  G  P  I  L  T  D  G  N  E  I

               535       545       555       565       575       585       595
526      AATGGGACTGATTTGGAAAGTGAGATGCATGAAGACACAGAAGAACTTAATGCCTTGCTGTACTCAGATGACGAG
176       N  G  T  D  L  E  S  E  M  H  E  D  T  E  E  L  N  A  L  L  Y  S  D  D  E

               610       620       630       640       650       660       670
601      AATGACTACTCTGAGGATGACGAAGAAACTAGCACCGGTCATTCGCCTAGTACAATGACATCTAACGAAAGATTG
201       N  D  Y  S  E  D  D  E  E  T  S  T  G  H  S  P  S  T  M  T  S  N  E  R  L

               685       695       705       715       725       735       745
676      AAAAGCTTTGAAGGAAGTGCTGAAGAAGTTGCTAGCACTGCTGAGCCAAGTAAAAAGCGAAAACTATCACTCATG
226       K  S  F  E  G  S  A  E  E  V  A  S  T  A  E  P  S  K  K  R  K  L  S  L  M

               760       770       780       790       800       810       820
751      GACACCGCAACTTCTAGGAAATCAATCCAGTTCTTGGGTTATGAAGAAGATGACGCAGAATCTAGCTGTGGTAAT
251       D  T  A  T  S  R  K  S  I  Q  F  L  G  Y  E  E  D  D  A  E  S  S  C  G  N

               835       845       855       865       875       885       895
826      GGCCTTAGTCAAGGTAATGGAGGGGAGGGCAGTAAAAGGCTGAGAAGGGAGAGAATTCGTGCAACTGTGAGCATT
276       G  L  S  Q  G  N  G  G  E  G  S  K  R  L  R  R  E  R  I  R  A  T  V  S  I

               910       920       930       940       950       960       970
901      CTACAGAGCATAATTCCTAATGGAATGGGAAAGGATGCAGTTGCGGTTCTTGATGAAGCTATTAACTACTTTAGA
301       L  Q  S  I  I  P  N  G  M  G  K  D  A  V  A  V  L  D  E  A  I  N  Y  F  R

               985       995      1005      1015
976      TCTTTGAAGCTCAAAGCCAAAGCTTTAGGACTTGATTCTCTCTAA
326       S  L  K  L  K  A  K  A  L  G  L  D  S  L  *
```

Figure S1. Nucleotide and deduced amino acid sequence of MfbHLH145.
